# Dynamized ultra-low dilution of *Ruta graveolens* disrupts plasma membrane organization and decreases migration of melanoma cancer cell

**DOI:** 10.1080/19336918.2022.2154732

**Published:** 2022-12-11

**Authors:** Camille Fuselier, Eleonore Dufay, Alexandre Berquand, Christine Terryn, Arnaud Bonnomet, Michael Molinari, Laurent Martiny, Christophe Schneider

**Affiliations:** a Center Armand-Frappier Santé Biotechnologie of the INRS, University of Quebec, Laval, Quebec, Canada; b CNRS UMR 7369 MEDyC, University of Reims Champagne-Ardenne, Reims, France; c LRN EA 4682, University of Reims Champagne-Ardenne, Reims, France; d Platform PICT, University of Reims Champagne-Ardenne, Reims, France; e Institute of Chemistry & Biology of Membranes & Nano-objects, Bordeaux, France

**Keywords:** Melanoma, dynamized ultra-low dilution, cancer, migration, plasma membrane

## Abstract

Cutaneous melanoma is a cancer with a very poor prognosis mainly because of metastatic dissemination and therefore a deregulation of cell migration. Current therapies can benefit from complementary medicines as supportive care in oncology. In our study, we show that a dynamized ultra-low dilution of *Ruta Graveolens* leads to an *in vitro* inhibition of migration on fibronectin of B16F10 melanoma cells, as well as a decrease in metastatic dissemination *in vivo*. These effects appear to be due to a disruption of plasma membrane organization, with a change in cell and membrane stiffness, associated with a disorganization of the actin cytoskeleton and a modification of the lipid composition of the plasma membrane. Together, these results demonstrate, in *in vitro* and *in vivo* models of cutaneous melanoma, an anti-cancer and anti-metastatic activity of ultra-low dynamized dilution of *Ruta graveolens* and reinforce its interest as complementary medicine in oncology.

## Introduction

Cell migration is a complex and heterogeneous process performed by all eukaryotic cell types. It is essential for the establishment of physiological functions such as immune surveillance, wound healing process or tissue morphogenesis during development [1]. The occurrence of a defect in these cell migration mechanisms is often deleterious to the organism or associated with severe pathologies such as cancer. In the context of cancer, the intervention of several parameters can contribute to the strategic choice of the mode of locomotion adopted by the tumor cells. Indeed, the composition, structure and rigidity of the extracellular matrix (ECM), together with the specific adhesive or proteolytic properties of each cell type, can modify their migration patterns. For example, excessive and abnormal migration of normally non-motile cells can be reactivated by a process of epithelial–mesenchymal transition and maintained by the overexpression of proteins regulating the actin cytoskeleton. The epithelial cell is then able to individualize by adopting the particular migratory characteristics of a mesenchymal cell, which are essential during tumor progression [2].

Cutaneous melanoma represents only 4% of skin cancers. Nevertheless, it becomes a major health problem when its prognosis is poor, since it causes 80% of deaths. The excessive proliferation of the primary tumor rapidly followed by metastatic dissemination allows cutaneous melanoma to be qualified as a very aggressive cancer, always difficult to treat. Indeed, the complexity of the tumor process, including, for example, a significant deregulation of cell migration, is still a major challenge to be understood in oncology today. Moreover, the level of adaptability and clonal heterogeneity of tumor cells allows them to establish a multitude of resistance mechanisms in order to evade conventional anti-melanoma therapies [3,4]. Only the combination of therapies targeting several features of melanoma cells, such as migration, invasion, proliferation, immune response or angiogenesis, can increase the chances of patient survival. In addition, conventional therapies very often cause severe adverse events leading patients to stop their treatment [5,6]. Therefore, it is more than essential to consider the development of complementary therapies in order to improve tolerance to the innovative treatments already on the market and adherence to those therapies with maintenance of the initial posology. This supportive care is part of a global approach to the patient, particularly with a view to ensure quality of life and benevolence [7]. Currently, about 20% of European oncology centers provide various forms of Complementary and Alternative Medicine (CAM) to their patients [8].

*Ruta graveolens* L. (Fetid Street) is a perennial herb with a woody stem, branching from the base, smooth and round, up to 80 cm high, belonging to the family *Rutaceae*. The plant is native to Europe, especially to the Mediterranean region, but it is widely distributed in all tropical and temperate regions. The main active substances of the medicinal plant *Ruta graveolens* are flavonoids [rutin; quercetin], furocoumarins and alkaloids [9–11]. Rutin, which is a phytochemical compound, has already shown its multiple pharmacological benefits including antioxidant, neuroprotective, cardioprotective, or even anticarcinogenic effects [12]. Interestingly, it has already been shown that this molecule, along with other flavonoids, was able to reduce the number of metastatic nodules in murine melanoma [13]. At high concentrations, however, this plant is associated with high toxicity on the functionality of multiple organs, particularly at the cardiovascular and hepatic level, sometimes even causing mortality and abortion [14]. *Ruta graveolens* extracts also demonstrate many biological properties such as cytotoxic activity on several human cancer cell lines, anti-tumor activity on animals, anti-inflammatory and anti-oxidant effects *in vivo* [15] So far, the main clinical indications for homeopathic dilutions of *Ruta graveolens* L. concern rheumatology and traumatology in case of sprains, tendonitis or low back pain.

Homeopathy, which is considered an integrative medicine, is undoubtedly one of the most widespread and frequently used supportive care treatments by patients. A descriptive study carried out in a French cancer ward between 2004 and 2005 on 195 patients, showed that 42% of Complementary and Alternative Medicine (CAM) users reported using homeopathy as an integrative medicine during their treatment [16]. Its use in oncology as a supportive care is relatively recent, and the number of clinical studies evaluating its beneficial effects is still insufficient. Nevertheless, it has been attested in the literature that its use does improve the quality of life of cancer patients (physical and mental fatigue), in addition to conventional treatments [17–19]. Similarly, a recent pilot study conducted clearly demonstrates the benefits of *Ruta graveolens* 5CH and *Rhus toxicodendron* 9CH in reducing joint pain and stiffness in breast cancer patients [20]. In addition, Frenkel *et al*. demonstrated the in *vitro* apoptotic mechanism of *Carcinosium* 30CH and *Phytollacca decandra* 30CH [21].

In recent years, many *in vitro* and *in vivo* publications have been published and begun to demonstrate the potential of homeopathic dilutions for affecting tumor progression. In the preclinical line of study, the influence of *Thuja occidentalis* mother tincture inhibited B16F10 melanoma cell-induced lung metastasis in C57BL/6 mice, while increasing the life span of the animals [22]. Similarly, MacLaughlin *et al*. were able to demonstrate a significant decrease in prostate tumor xenografts with the use of *Sabal serrulata* [23]. Finally, numerous studies have demonstrated the role of different homeopathic strains in modulating the cancer immune system. Among them, the use of *Calcarea carbonica* 6CH enhanced apoptosis of murine sarcoma and carcinoma cells by enhancing the activity of T cells that were immunosuppressed by the tumor [24]. In their first publication, Arora *et al*. demonstrated the anti-proliferative and cytotoxic effects of mother tinctures and several dilutions of *Ruta graveolens* on colonic cancer cells (COLO-205) and *Phytolacca decandra* on breast cancer cells (MCF-7) [25]. Finally, further study showed that the mother tincture and 30CH dilution of *Ruta graveolens* showed the best effects against COLO-205 cell survival. Nuclear changes related to increased fragmented DNA and increased expression of specific genes such as caspase 3, Bax or p21, indicate that *Ruta graveolens* induces cancer cell death by apoptosis [26]. We have recently demonstrated in our laboratory that *Phenacetinum* 4CH altered both the *in vitro* tumor progression of melanoma but also slowed down tumor development and angiogenesis *in vivo* [27,28]. In this study, we describe the effects of *Ruta graveolens* 9CH on plasma membrane re-organization and its influence on melanoma cell migration.

## Materials and methods

### Cells lines and reagents/antibodies

Murine melanoma cell line B16F10 was obtained from ATCC. Cells were cultured in RPMI 1640 (Gibco) containing 10% fetal bovine serum (FBS, from ATCC) in standard conditions (37°C, 5% CO_2_). Cell line was used at low passage number (<15) and was mycoplasma free (MycoAlert; Lonza). *Ruta graveolens* 9CH was obtained from Boiron Laboratories (Messimy, France). The mother tincture (MT) was produced based on the 1.1.10 (2371) method of the European Pharmacopoeia (Ph. Eur.) guidelines. The 9CH dilution was obtained from successive centesimal dilutions in sterile water (OTEC, Aguettant France) starting with MT and always followed by vigorous mechanical shaking. The vehicle control is sterile water (OTEC). For each experiment, cells were blindly treated with 5% of this dilution or control in RPMI 1640 0.5% FBS. Fibronectin, Mitomycin C and Laurdan were obtained from Sigma Aldrich (France). The probes used in this study are an anti-phalloidin coupled to Alexa-fluor 488 (Invitrogen), an anti-pip2 antibody (ab11039, Abcam).

### Migration into dispersed cells assay

Cells were seeded at an initial density of 7.5 × 10^3^ cells/well in a 24-well culture plate coated with fibronectin (7 μg/mL) for 24 h. Cells were treated with mitomycin C [1 μg/mL] for 2 h. Then, they were rinsed with RPMI 1640 0.5% FBS and treated with *Ruta graveolens* 9CH or control in the same medium. Immediately, the plate was placed in the thermostatically controlled chamber, enriched with 5% CO_2_ of the microscope (AxioObserver Z1, Zeiss). Images were acquired every 10 min during 24 h. For each experiment, 60 random cells were tracked with Manual Tracking plugin of ImageJ. The plot representation was obtained with Chemotaxis and Migration tool plugin (Ibidi, free download).

### Transwell migration assay

A 24-well transwell chamber (Greiner Bio-One, Dutscher, France) with an 8-μm pore PET membrane was used to perform the migration assay and coated with fibronectin at 7 μg/mL. The lower chamber was filled with 600 μL RPMI 1640 conditioned medium (made with NIH3T3 cell line). Then, 200 μL B16F10 melanoma cell suspension (2.5 × 10^5^ cells/mL with 0.5% FBS), containing 5% of *Ruta graveolens* 9CH or control, was added to the insert. The cells were allowed to migrate at 37°C with 5% CO2 over 6 h. The inserts were washed in PBS and fixed with methanol for 15 min. Non-migrating cells were removed from the upper surface of the inserts by gently scrubbing with a cotton-tipped swab. Each PET membrane was cut and stained with mounting medium DAPI (ProLong Gold DAPI, ThermoFischer), between blades and slats. For counting, 10 random pictures were taken per membrane, and each condition was made in duplicate. Experiments were performed at least in triplicate.

### Cell circularity

Cell morphology was quantified using circularity index on ImageJ software by the following formula: 4π(area)/(perimeter)^2^. This formula gives a circularity index ranging from 0 at 1, where value 0 corresponds to an elongated shape and value 1 to a rounded morphology. Cells were chosen randomly from tracking obtained previously by time-lapse movies, and the average of the contours was made. Experiments were performed at least in triplicate.

### Atomic force microscopy

The AFM utilized in this study is the Bioscope CatalystTM (Bruker, Billerica, USA) coupled to a Nikon Eclipse Ti inverted microscope (Nikon, Tokyo, Japan). The glass-bottom petri dishes (50 mm in diameter, WillCo Wells, Amsterdam, the Netherlands) were put onto the AFM stage and observed with Bright Field illumination in order to locate the cells. One day prior to the experiments, cells were seeded at a density of 5 × 10^4^ cells/mL on the plates previously coated with fibronectin (7 μg/mL). After a 24 h period, the cells were rinsed twice with medium low serum and treated with 5% of *Ruta graveolens* 9CH or control in RPMI 0.5% FBS, for 1 h at 37°C, 5% CO_2_. All images were captured in Peak Force Quantitative Nanomechanical Mapping (PFQNM) mode. The Young’s moduli were calculated by using a Sneddon fit. For PFQNM experiments, we used a PeakForce frequency of 0.25 kHz in order to maximize the contact time between the tip and the sample. The PeakForce amplitude was set at 1 μm. The loading force was lowered down to a few tens of pN to avoid generating mechanical stress of the cells. Images were captured randomly in culture medium at a resolution of 256 or 128 pixels per line, at 37°C using a Perfusing Stage Incubator. Regarding the Young’s modulus calculation, a minimum of three analyses on three different cells (perinucleus areas were avoided) were performed and the experiments were triplicated for each sample type.

### Immunofluorescence staining

B16F10 were plated at low density onto plastic LabTek (Nunc, Dutscher, France) previously coated with 7 μg/mL of fibronectin for 12 h at 4°C and blocked with 1% BSA. Cells were incubated on the LabTek for 24 h at 37°C in 5% CO_2_ to allow spreading. Subsequently, the cells were treated with 5% *Ruta graveolens* 9CH or control in medium low serum during 1 h at 37°C and 5% CO_2_. Actin staining: cells were rinsed once gently with medium low serum at 37°C and once with glutaraldehyde 0.1% in PBS. Cells were fixed with glutaraldehyde 0.5% for 10 min at room temperature (RT). After two rinses with PBS, the cells were blocked with 10% BSA for 1 h at RT. Cells were incubated with Alexa FluorTM 488 Phalloidin probe for 1 h, in 2% BSA/TBS-Triton X100 (1:100 dilution). After incubation, LabTek were washed in 0.1% Triton/TBS and mounted onto slides using mounting medium DAPI. Random observations of LabTek were taken with a fluorescent microscope (BX51WI, Olympus) at Ex/Em = 493/517 nm.

### Cholesterol staining and quantification

B16F10 were plated on fibronectin and incubated in LabTek at 37°C in 5% CO_2_. Subsequently, the cells were treated with 5% *Ruta graveolens* 9CH or control in medium low serum during 1 h. Cholesterol assay kit (Abcam #ab133116) was used to stain cholesterol with Filipin III. For the cholesterol quantification, lipids were extracted after cells treatment with 200 µl of chloroform/isopropanol/NP40 (7/11/0.1, v/v/v). The free cholesterol part was measured with the Cholesterol Assay Kit (Abcam #65359) according to the manufacturer's instructions.

### Laurdan two-photon microscopy

Laurdan is an amphiphilic fluorescent probe able to penetrate a biological membrane, and to detect changes in membrane phase properties through its emission spectral shift. B16F10 were plated at low density onto a cell culture dish (35 mm diameter, FluoroDish WPI), previously coated with 7 μg/mL fibronectin for 12 h at 4°C and blocked with 1% BSA. At the end of the day, cells were rinsed twice with medium low serum, then 1.9 ml of this medium with 2 μL of Laurdan (2-dimethylamino(6-lauroyl)naphthalene) at [5μM] were added overnight at 37°C. In the following morning, 100 μL of drug was added directly into a well, and around 10 cells were imaged with confocal microscope (LSM 710 NLO ZEISS). Laurdan intensity images were randomly recorded simultaneously with emission in the range of 400–460 nm and 470–530 nm. Membrane fluidity was measured in terms of ratio of emission intensities by using Generalized Polarization (GP) value, defined as GP = I (400 − 600) − GI (470 − 530)/(400 − 460) − GI (470 − 530).

## Calcium flux analysis

The cells were seeded in 2-well Labteks (153,580, Fischer Scientific), previously coated with fibronectin. The next day, the cells were rinsed with RPMI 0.5% SVF medium. The cells were then incubated for 30 min at 37°C with 750 μl of RPMI 0.5% SVF medium and 750 μl of the Fluo-4 Direct Calcium Assay kit solution (Thermo Fisher). After this incubation time, the cells were placed under the fluorescence microscope. Images are taken every second. *Ruta graveolens* 9CH or control at 5% and ionomycin (#10134232, Fisher Scientific) [10 μM] are directly injected into the well during acquisition at 10 seconds. The fluorescence intensity of 10 random cells per condition is analyzed using the macro-trace–intensity-modif-modif-fluo4-multiROI by C. Terryn.

## *In vivo* metastasis model

The animal study was reviewed and approved by the University of Reims Champagne-Ardenne (CEEA-RCA n°56) and the CNRS (Center National de la Recherche Scientifique). For studies on a lung metastasis model, a suspension of B16F10 cells (2.5 × 10^5^) was injected into the tail veins of female C57BL/6 mice. Each group contains at least eight mice. Each mouse was randomly allocated to the different groups. Mice were daily and blindly treated with 100 µl *Ruta graveolens* 9CH or control (OTEC) for 15 days. We used ‘test’ mice to determine the date of sacrifice of the mice according to the number of pulmonary nodules observed in them. After dissection, isolation and photography of the lungs, the number of lung nodules was counted.

## Statistical analysis

GraphPad Prism 8.4.0 software (GraphPad, La Jolla, CA) was used for all statistical analyses. Each result is representative of at least three independent experiments. Data are expressed as the mean ± SEM. Significance was assessed using Student’s t-test or One Way ANOVA for Gaussian distribution of data parametric tests, and Mann-Whitney U test was used for others (non-parametric test). For all tests, statistical significance was assumed when p < 0.05 (*).

## Results

### Ruta graveolens *9CH decreases B16 cells stiffness*

**Figure 1.**
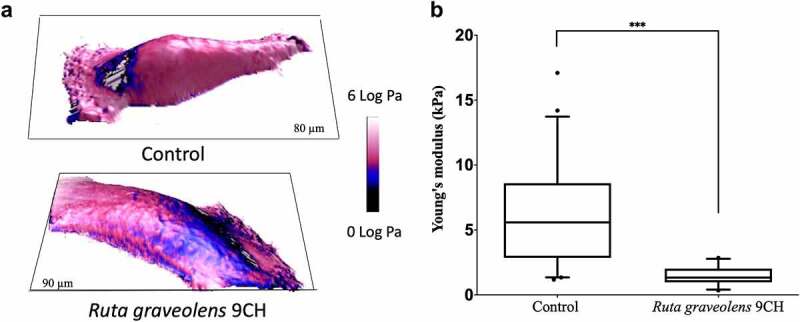
Influence of *Ruta Graveolens* 9CH on cell stiffness of living individual B16F10 cells.

The 3D topographic images obtained by the AFM technique show that *Ruta graveolens* 9CH decreases B16F10 cell stiffness (Figure 1a). Analyses were made on perinuclear areas, to get rid of the nucleus and membrane extensions which will lead to artifacts in the mechanical measurements. For the different conditions, Figure 1a shows the topography of the cells in 3D, while the color code is linked to the values of the reduced Young’s modulus (elasticity modulus). After 1 h, B16F10 treated cells showed a significant decrease in Young modulus (characterized by blue color instead of the pink one for the non-treated cells). Indeed, Young’s modulus analysis confirms that *Ruta graveolens* 9CH decreases the stiffness of B16F10 cells by 3.9-fold (average value of 5.9 ± 3.7 kPa for control cells and 1.5 ± 0.7 kPa for treated cells) (Figure 1b). In addition, and as already shown with *Phenacetinum* 4CH [27], *Ruta graveolens* 9CH did not alter normal MEFs cell stiffness (data not shown). As a conclusion, these results indicate that *Ruta Graveolens* 9CH is able to specifically decrease the overall stiffness of the cellular envelope in B16F10 cells.

Determination of cell stiffness was conducted with Atomic Force Microscopy (AFM) by Peak Force Quantitative Nanomechanical Mapping measurements (PFQNM mode). After 1 h of treatment with 5% of *Ruta graveolens* 9CH or control, cell stiffness of dispersed living B16F10 cells, seeded on fibronectin, was studied for their elastic properties (Pa). (a) Topographic images are represented in 3D, while the color code is linked to the cell Young’s modulus values (log Pa unit) according to the experimental conditions. Both are representative of the cell behavior for each condition. (b) The average values of Young’s Modulus on perinuclear areas are represented in box and whiskers, n = 3, ***p < 0.001. Data are expressed as means ± SEM.

### Ruta graveolens *9CH modifies the phospholipid organization*

**Figure 2.**
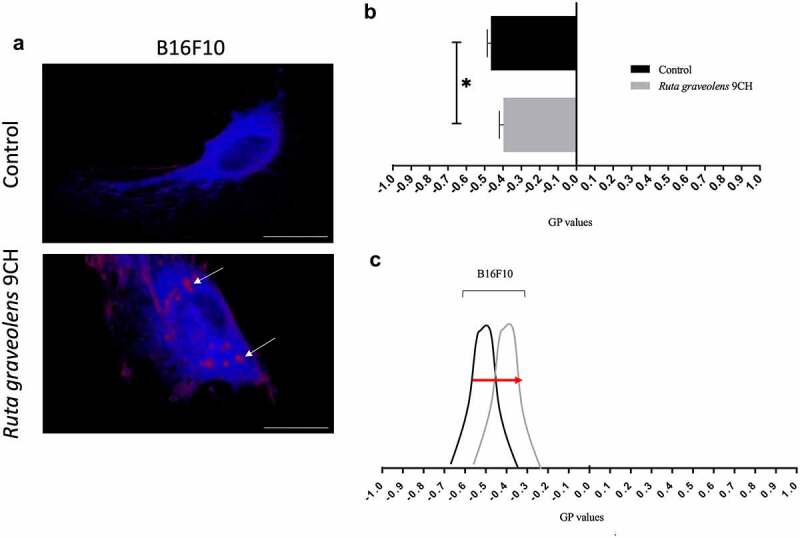
Effects of *Ruta Graveolens* 9CH on phospholipid membrane organization.

In order to reinforce the idea that *Ruta Graveolens* 9CH disrupts the organization of the overall cellular envelope, we studied its action on the phospholipid organization contained in the plasma membrane of cells plated on fibronectin. The data presented on Figure 2 show a different distribution of fluorescence depending on conditions after 1 h of treatment. Results indicate (Figure 2a) the distribution between disordered lipid phases (Ld, in blue fluorescence) and ordered lipid phases (Lo, in red fluorescence). In B16F10 control cells, a majority of Ld phases are noticed whereas in *Ruta Graveolens* 9CH treated cells, large and localized Lo domains are present (white arrows, Figure 2a). We quantified the membrane lipid order and the histogram presented in Figure 2b indicates generalized polarization (GP) values of perinuclear areas. In control, we noticed that B16F10 had an average GP value of −0.47 ± 0.02. *Ruta Graveolens* 9CH treatment causes an increase in GP values of −0.39 ± 0.02 for B16F10 cells. To conclude, *Ruta graveolens* 9CH significantly increases after 1 h of treatment the ordered lipid phases by 11% for B16F10 cells compared with control and increase the rigidity and lipid packing of the bilayer membrane (Figure 2c).

B16F10 cells were seeded on fibronectin, incubated with Laurdan [5 μM] overnight at 37°C and then treated with 5% homeopathic drug or control. After 1 h, living cells were excited at 800 nm with a bi-photon confocal microscope, immersion objective 60× (scale bar, 10 μm). The camera then captures two simultaneous images with wavelengths range to 400–460 nm (liquid-ordered phase ‘lo’, in red) and 470–530 nm (liquid-disordered phase ‘ld’, in blue). (a) Representative confocal microscopy images according to experimental conditions where white arrows show ordered lipid phases. (b and c) Intensity of pixels was introduced in generalized polarization equation that provides GP values for *Ruta graveolens* 9CH and control conditions as measurements of membrane fluidity. Quantification of GP values are ranged from −1 (more fluid) to +1 (more gel), n = 3, *p < 0.05. Data are expressed as means ± SEM.

### Ruta graveolens *9CH changes the distribution of membrane cholesterol in B16 cells*

**Figure 3.**
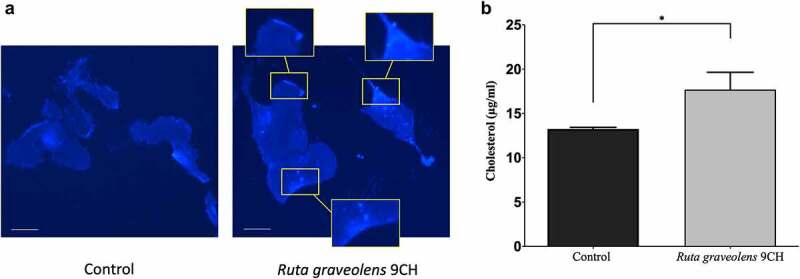
Effects of *Ruta Graveolens* 9CH on cholesterol.

Following the results of generalized polarization that show an increase in ordered lipid domains and therefore probably an increase in bilayer membrane stiffness in the presence of *Ruta graveolens* 9CH, we wanted to confirm these data by directly detecting the presence of cholesterol at the plasma membrane of B16F10 cells using the fluorescent marker Filipin. The results are shown in Figure 3. In control, cholesterol locates mainly on peri-nuclear areas of B16F10 cells (Figure 3a Left). A treatment with *Ruta graveolens* 9CH allows to observe an accumulation of cholesterol homogeneously distributed on the whole plasma membrane in the form of large clusters (3a Right). The analysis of fluorescence intensities allowed us to highlight a very marked enrichment of membrane cholesterol when B16F10 cells are treated with *Ruta graveolens* 9CH (3a Right boxes), which could promote the increase in ordered lipid phases observed previously. Numerous scientific studies attest that free cholesterol (as opposed to esterified cholesterol) resides mainly within plasma membranes [29,30]. To independently confirm the previous results, we quantified the free cholesterol level after lipid extraction on B16F10 cells. The amount of free cholesterol is about 13.3 μg/mL in control B16F10 cells. However, treatment with *Ruta graveolens* 9CH significantly increases the amount of free cholesterol compared to control. Indeed, the free cholesterol concentration reaches 17.7 μg/mL at 1 h (+25%) for B16F10 cells treated with *Ruta graveolens* 9CH (Figure 3b). Like those obtained by fluorescence on phospholipid organization at cell membrane, these results also show that membrane cholesterol levels increase in the presence of *Ruta graveolens* 9CH.

(a) Representative images showing membrane cholesterol in Filipin-labeled B1610 cells (Obj ×20, scale bar, 10 μm). (b) Quantification of the amounts of free cholesterol fraction of B16F10 cells treated with 5% *Ruta graveolens* 9CH or control. Results are expressed as free cholesterol concentration (μg/ml). Means ± SEM of results are plotted, n = 3, *p < 0.05.

### Ruta graveolens *9CH disrupts induced calcium flows*

**Figure 4.**
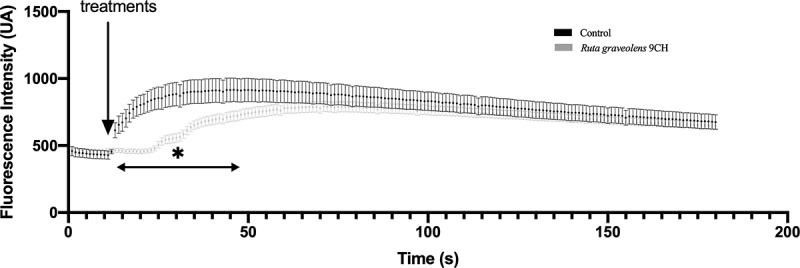
Effects of *Ruta Graveolens* 9CH on induced-calcium flows.

To reinforce the idea of a change in plasma membrane reorganization, we looked at the effect of *Ruta graveolens* 9CH on ionomycin-induced calcium flux. Ionomycin is a calcium ionophore capable of increasing intracellular calcium by facilitating its transport across the plasma membrane via activation of calcium channels. For this purpose, B16F10 cells were treated simultaneously with ionomycin and *Ruta graveolens* 9CH. The results presented in Figure 4 show a significant decrease in the intensity of calcium flux as well as a delayed response under the effect of *Ruta graveolens* 9CH. This result therefore tends to support the idea of a disruption and a reorganization of the plasma membrane under the effect of treatment.

B16F10 cells were seeded in 2-well Labteks on fibronectin. The cells were incubated with Fluo-4 and placed under a fluorescence microscope. *Ruta graveolens* 9CH or control (5%), and ionomycin were directly injected into the well at 10 seconds during a real-time acquisition of 200 s. The fluorescence intensity of 10 random cells per condition was analyzed using the macro-trace–intensity-modif-modif-fluo4-multiROI. Data are expressed as means of Arbitrary Unit (AU) of fluorescence intensity ± SEM, n = 3, *p < 0.05.

### Ruta graveolens *9CH alters the structure of actin filaments in B16F10 cells*

**Figure 5.**
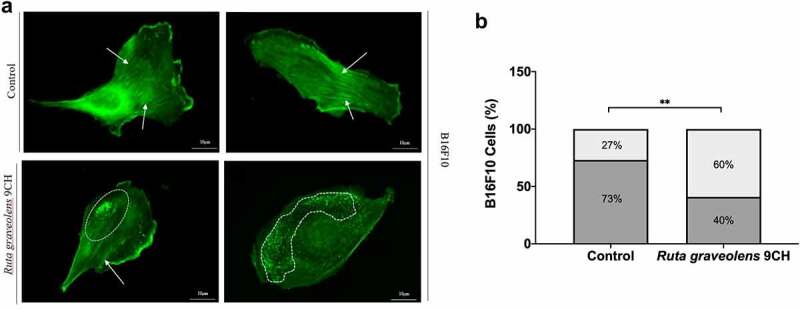
Effects of *Ruta Graveolens* 9CH on actine organization.

These data on membrane reorganization were completed by the study of actin filaments by immunofluorescence since cytoskeleton as plasma membrane is part of the cellular envelope. To confirm the results obtained on cellular envelope stiffness, we quantified the characteristic actin profiles found in each condition (Figure 5). As a reminder, most of the control cells have a highly organized actin cytoskeleton profile composed of numerous intact actin filaments (Figure 5a top, white arrow). In contrast, the treatment of the cells with *Ruta graveolens* 9CH causes a significant destructuring of this actin cytoskeleton (Figure 5a bottom, dotted white circle). These two specific phenotypes were therefore counted. After 1 h of treatment, 73% of the B16F10 control cells show an organized cytoskeleton in the form of stress fibers and 27% of them have a disorganized cytoskeleton (Figure 5b). In general, the majority of the B16F10 control cell population maintains an organized actin network over time. However, when these same cells are treated with *Ruta graveolens* 9CH, there is a significant increase in cell profiles where actin is disorganized. Indeed, the presence of a disorganized cytoskeleton is doubled after 1 h of treatment with *Ruta graveolens* 9CH for B16F10 cells (from 27% of disorganized actin for control cells to 60% for treated cells) (Figure 5b). These analyses allow us to confirm that the homeopathic treatment accelerates the destruction of the actin network of B16F10 cells that confirms the decrease in cellular envelope rigidity observed previously.

B16F10 cells were seeded on fibronectin and treated for 1 h with 5% *Ruta graveolens* 9CH or control. Cells were fixed with glutaraldehyde, permeabilized with TritonX-100 and stained with Phalloïdin-Alexa Fluor 488 (actin filament in green). (a) Representative cells treated with control (top) or Ruta graveolens (bottom) stained with Phalloidin and visualized with an epifluorescence microscope, objective 20× (scale bar, 10 μm). The white arrows show actin stress fibers, and dotted white circles show a disruption of cortical actin. (b) Quantified profile types like stress fibers organized (in gray) or disorganized (light gray) actin networks in each condition and representative of presented data using Image J software. They are expressed in means normalized in percentage ± SEM, n = 3 where 60 random cells were analyzed per condition and experiment, **p < 0.01.

### Ruta graveolens *9CH decreases 2D and 3D dispersed cells migration and increases B16F10 cells circularity*

**Figure 6.**
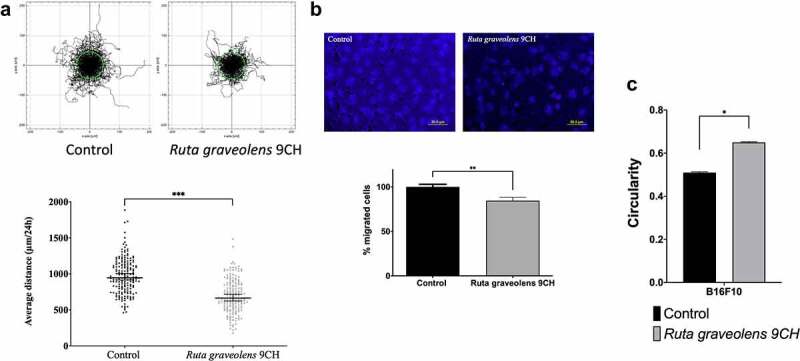
Effects of *Ruta Graveolens* 9CH on B16F10 2D dispersed migration, 3D migration and circularity.

To investigate the consequences of cellular envelope reorganization, we studied the effects of *Ruta graveolens* 9CH on B16F10 cell migration. Pictures in Figure 6a represent 180 trajectory profiles taken randomly and blindly depending on the following conditions. The initial position of each cell was set at the origin (0,0) of coordinates. Under these conditions, representative tracks showed that among the 50% of cells outside the circle in control situation, only 33% were out when they were treated with *Ruta Graveolens* 9CH (Figure 6a; top). Thus, the diminution between the control cells and the treated cells outside circles was 34% for B16F10 cells. Moreover, *Ruta graveolens* 9CH significantly decreased the 24 h average 2D migration distance of isolated cells by 30% for B16F10 cells (average distance traveled by control cells 945 μm and 664 μm for treated cells) (Figure 6a; bottom). Similarly, this treatment significantly decreased 3D migration by 26% for B16F10 cells (Figure 6b). At last, *Ruta graveolens* 9CH significantly increased the circularity of B16F10 cells by 22% 24 h after treatment (Figure 6c). Taken together, these results show that *Ruta graveolens* 9CH decreases the migratory capacity of B16F10 cells presumably by altering the organization of the cellular envelope both on plasma membrane and cytoskeleton actin filaments.

(a) For 2D dispersed migration, B16F10 cells were seeded on fibronectin, treated with 5% of *Ruta graveolens* 9CH or control and random cells were tracked for 24 h using Manual Tracking plugin. Data obtained were analyzed by chemotaxis plugin (trajectories of 60 random cells per image) on ImageJ software, and quantification of average distance traveled in 24 h was expressed in μm, n = 3. (b) For 3D cell migration, B16F10 cells were seeded on Boyden chamber coated with fibronectin, treated with 5% of *Ruta graveolens* 9CH or control and allowed to migrate for 6 h. Percentage of nuclei (under the polycarbonate membrane) was counted by ImageJ software, n = 3 (scale is 50 μm). (c) The average cell circularity (from 0 to 1 in the sense of circularity) was studied with ImageJ from 60 random cells from 2D dispersed migration at 24 h, n = 3. Data are expressed as means ± SEM. *p < 0.05, ***p < 0.01, ***p < 0.001.

### Ruta graveolens *9CH decreases B16F10-induced lung metastases and improves survival*

**Figure 7.**
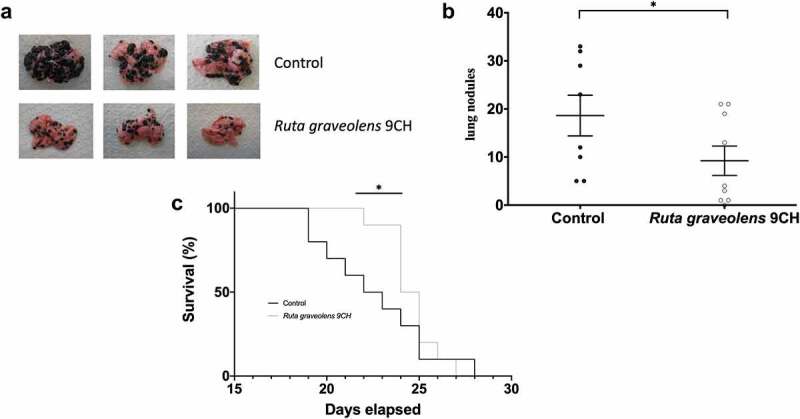
Effects of *Ruta Graveolens* 9CH on *in vivo* lung metastasis and survival.

As tumor cell migration is one of the major events in the development of metastasis, we studied the anti-metastatic activity of *Ruta graveolens* 9CH in a mouse model of lung metastasis. After injection of 2.5 × 10^5^ invasive B16F10 melanoma cells into the tail vein of mice, the animals were treated daily and blindly by intraperitoneal injection of *Ruta graveolens* 9CH or control until sacrifice. The results are shown in Figure 7. After 15 days of treatment, representative lungs were collected and photographed (Figure 7a) and we could observe that *Ruta graveolens* 9CH treatment significantly reduced the number of metastases. The quantification of these metastases showed a 50% decrease in the treated mice (Figure 7b). In turn, the overall survival of treatment-mice was significantly improved in the early days (40% at D22 and 55% at D23) of mortality (Figure 7c). In view of the previous results, it is quite possible to attribute this decrease to the loss of motility of B16F10 cells.

B16F10 cells were injected into the tail vein of mice at J0. (a) Representative images of the lungs of *Ruta graveolens* 9CH-treated and untreated mice after 15 days. (b) Quantification of the number of lung nodules in both experimental conditions. (c) Kaplan–Meier survival curve with control (black) and *Ruta graveolens* 9CH (gray). Data are expressed as means ± SEM. *p < 0.05.

## Discussion

Cutaneous melanoma is the most aggressive skin cancer. Surgery is still the best treatment today, but it has a very poor prognosis when it becomes metastatic [4,31–34]. In this work, we studied cell migration, which is an important mechanism involved during cancer progression. Although the use of *Ruta graveolens* is common in cases of rheumatology or joint pain, the characterization of its effects on the mechanical properties of cells is poorly referenced [35]. Therefore, this study examined the effect of *Ruta graveolens* 9CH on highly invasive melanoma cells cultivated on fibronectin matrix. Thanks to the use of multidisciplinary approaches, our expertise has enabled us to characterize and attribute new anti-cancer cell properties to *Ruta graveolens* 9Ch on *in vitro* and *in vivo* models.

The cell is a living system that can move independently, partly thanks to the extracellular matrix, using metabolic energy to perform mechanical work. Tumor cells also have a set of particular mechanical properties in order to survive and complete the metastatic dissemination through tissues. Cell stiffness is one property often highlighted in scientific reports, thanks to its strong correlation with cell motility [36–39]. Based on a 2D model with fibronectin, we placed our study context in a system allowing the melanoma cells to generate enough traction forces through their adhesion points with their matrix environment and make them move forward efficiently. Indeed, the more or less rigid composition of tumor microenvironment constitutes a decisive elastic substrate that strongly influences the cancer cells aggressiveness and deserves to be taken into account on *in vitro* studies [36,40].

The literature of a large panel of studies shows that plasma membrane holds a critical place in maintaining cellular homeostasis [41–44]. Despite its structural function, the plasma membrane supports important signaling functions through receptors, enzymatic activities, or mechanisms of endocytosis and exocytosis. Together, these functions govern a communication between the cell and its environment, which is also essential for directing cell migration [44]. Besides the lipid composition and organization of plasma membrane, it regulates the physical properties of the underlying cytoskeleton. Among existing lipids, one of the major components of mammalian cell membranes is cholesterol [45–47]. Because of its rigid planar four-ring nucleus, cholesterol plays a crucial support role in the plasma membrane structure and directly affects its fluidity. Obviously, an abnormal change in membrane fluidity can have important repercussions on cell function such as: cell deformability, membrane trafficking, ion or nutrient transport, and receptors or even on enzyme activity. In the context of cancer, it has already been assumed that a very low concentration of membrane cholesterol allowed cell membranes to be more easily deformed and more fluid, increasing also the metastatic capacity of cells [48–51]. Conversely, under the action of *Ruta graveolens* 9CH, our results showed a rapid and significant accumulation of membrane cholesterol on the upper surface of B16F10 cells. Studies report that the hydrophobic nature of cholesterol allows is a factor allowing it to interact predominantly with the long acyl chains of sphingolipids located at the core of the phospholipid bilayer to form lipid rafts [45,52,53]. In fact, the cell plasma membrane has a structure of disordered membrane areas Ld (absence of rafts) punctuated by more ordered domains Lo (presence of rafts) [54,55]. To illustrate this relationship, Ermilova *et al*. demonstrated for example through molecular dynamics simulations, that the addition of cholesterol molecules within Ld membrane phases would increase the order of these acyl chains and thus the thickness of the plasma membrane through a capacitor effect [56]. Hence, an enrichment of membrane cholesterol could have a global effect on plasma membrane structure and function, particularly essential for the formation of lipid rafts and their gathering as macrodomains [57,58]. In this sense, we have indeed shown that *Ruta graveolens* 9CH was able to enrich the plasma membrane of B16F10 cells with ordered lipid domains (Lo). This suggests that the accumulation of cholesterol in the plasma membranes of melanoma cells induced by *Ruta graveolens* 9CH treatment may be involved in the formation of large ordered lipid phases, which most likely increases their rigidity and packing. Literature reports that an increase in membrane stiffness would also be able to generate permeability defect and specially on membrane ion channels. Membrane permeability is essential to the passive transmembrane transport of small molecules [59,60]. There is conflicting evidence regarding enhancement or limitation of membrane permeability following the accumulation of lipid rafts by the presence of cholesterol into the lipid bilayer [60,61]. In any case, the alteration of membrane permeability inevitably leads to a disruption of the flow of essential chemical molecules. We have shown that under the action of *Ruta graveolens* 9CH, the intracellular calcium gradients weaken rapidly after the treatment. Levitan *et al*. reviewed the role of cholesterol and lipid rafts in the regulation of the major types of ions channels, implying that some calcium channels are strongly inhibited by cells membranes enriched by cholesterol [62]. It would therefore be consistent that an increase of membrane cholesterol domains observed under the action of *Ruta graveolens* 9CH could contain calcium channels, and block calcium influx into the cell. Moreover, Wei *et al*. provide an additional fact that the calcium concentration is greatest at the back of a polarized cell, reducing as one moves toward the front of the cell [63]. The visible packing of ordered liquid phase observed on the upper central part of B16F10 cells with *Ruta graveolens* 9CH could also impact the localization of calcium channels that should initially be located at the front of the cell. Although the analysis deserves further investigation, we can deduce that the spatiotemporal regulation of calcium signaling is altered due to the particular structural conformation of cholesterol clustering under *Ruta graveolens* 9CH treatment.

Cell elasticity is also controlled by the cooperation between plasma membrane and intracellular cytoskeleton. Together, they confer to the cell the essential aspects of its architectural properties and work in a coordinated way to ensure, among other things, an effective migration. So, in addition to stabilizing with each other through lateral interactions, rafts can also activate and stabilize through the underlying cortical actin network that binds to plasma membranes [57,58]. Indeed, some studies show that rafts are particularly rich in cytoskeletal proteins, nicely characterizing the cytoskeleton as an important scaffolding element for the organization of plasma membranes [64,65]. While we supposed that *Ruta graveolens* 9CH increased lipid rafts of B16F10 cells through increasing cholesterol level, we also showed that homeopathic treatment destructed their filamentous actin network. As we did, the investigations of Sun *et al*. also described that cholesterol enrichment also decreased membrane-cytoskeleton adhesion, whereas cholesterol depletion had the opposite effect [66]. These results therefore open a new window of discussion by reconsidering the idea often established, according to which the formation of lipid rafts will necessarily serve as the focal points of the actin cytoskeleton. Furthermore, it is precisely the polymerized cortical actin adhered to the plasma membrane that reflects the level of cellular rigidity. It perfectly explains our AFM results demonstrating that *Ruta graveolens* 9CH drastically decreases the overall cellular envelope rigidity of B16F10 cells by disturbing actin cytoskeleton. Some recent studies still support our results by also showing the existence of an inverse relationship between plasma membrane rigidity and cell fluidity. For example, Wu *et al*. demonstrated that cholesterol-depletion-induced raft disruption caused a significant increase in stiffness on HUVECs cells [67]. Levitan *et al*. also demonstrated that the removal of cholesterol from the plasma membrane of endothelial cells induced an increase in cell stiffness and at the same time led to its fluidization [68]. To ultimately understand the effects of *Ruta graveolens* 9CH, we clearly showed that homeopathic dilution disturbed highly B16F10 cell polarity and migration. Together, these results clearly converge to show that *Ruta graveolens* 9CH seriously affects the integrity of melanoma cell structure and their function, probably by disrupting the plasma membrane and/or cortical cytoskeleton to finally decrease the melanoma cell migration.

Melanoma is known to be one of the most frequently metastasizing malignant tumors and for which cell migration is a necessary process. Metastatic cancer cells are usually softer and more highly deformable than healthy cells, which represents a significant advantage for their invasive potential [36,37,69]. However, this evidence stays debatable, since some researchers present contradictory results with cancerous and invasive cells being more rigid than healthy cells [70]. Ultimately, we demonstrated *in vivo* that our highly diluted molecule, like other flavonoids [13], was able to reduce the number of pulmonary metastatic nodules of murine melanoma B16F10 cell line by drastically reducing their cellular rigidity. On the contrary, authors Watanabe *et al*. showed that (-)-epigallocatechin gallate-B16F10 treated cells reveal a targeted increase in nuclear cell stiffness with an inhibition of cell migration [38]. This information reveals the obvious complexity of the cellular machinery and its environment. To sum up, a very low or very high cell stiffness probably disrupts the migratory capacity of cells. Therefore, cell stiffness should be a parameter to be considered in cancer treatment since it appears as a key point in the ability of cells to metastasize.

In conclusion, all these results have allowed us to highlight new anti-melanoma biological characteristics of *Ruta graveolens* 9CH. Overall, *Ruta graveolens* 9CH slows down the migration of B16F10 cells isolated *in vitro* by a sequence of mechanical alterations following different chronological events. We assume that it acts initially on the plasma membranes of the cells. Probably due to a mechanical effect still unknown, it disturbs the organization of phospholipids through the accumulation of cholesterol which could be the cause of the blocking of intracellular calcium influx. These ordered lipid phases, often associated with lipid rafts, are themselves known to be signaling platforms considerably involved in cell migration. Interestingly, *Ruta graveolens* 9CH also induces a significant destruction of actin filaments. All these phenomena lead to a loss of B16F10 cell polarity and an increase in their circularity, which inevitably decreases directed cell migration. This last process is also a key step for metastatic dissemination. The decrease in the number of pulmonary nodules observed in mice treated with *Ruta graveolens* 9CH could be correlated with the disruption of B16F10 cells migration observed *in vitro*. Thus, *Ruta graveolens* 9CH seems to have visible anti-cancer triggering mechanisms on *in vitro* melanoma cells and in a mouse model of metastasis, which constitutes an interesting avenue of research that deserves to be refined.

## Data Availability

The data that support the findings of this study are available from the corresponding author upon reasonable request
